# Effects of Adipose-Derived Biogenic Nanoparticle-Associated microRNA-451a on Toll-like Receptor 4-Induced Cytokines

**DOI:** 10.3390/pharmaceutics14010016

**Published:** 2021-12-22

**Authors:** Xinghua Wang, Anthony Pham, Lu Kang, Sierra A. Walker, Irina Davidovich, Dalila Iannotta, Sarvam P. TerKonda, Shane Shapiro, Yeshayahu Talmon, Si Pham, Joy Wolfram

**Affiliations:** 1 Department of Biochemistry and Molecular Biology, Mayo Clinic, Jacksonville, FL 32224, USA; wang.xinghua@mayo.edu (X.W.); pham.anthony@myao.edu (A.P.); walker.sierra1@mayo.edu (S.A.W.); iannotta.dalila@mayo.edu (D.I.); 2 Department of Cardiothoracic Surgery, Mayo Clinic, Jacksonville, FL 32224, USA; kang.lu@mayo.edu; 3 Department of Chemical Engineering and the Russell Berrie Nanotechnology Institute (RBNI), Technion-Israel Institute of Technology, Haifa 3200003, Israel; irinad@campus.technion.ac.il (I.D.); ishi@technion.ac.il (Y.T.); 4 Department of Plastic Surgery, Mayo Clinic, Jacksonville, FL 32224, USA; terkonda.sarvam@mayo.edu; 5 Center for Regenerative Medicine, Mayo Clinic, Jacksonville, FL 32224, USA; shapiro.shane@mayo.edu; 6 Department of Orthopedic Surgery, Mayo Clinic, Jacksonville, FL 32224, USA; 7 Department of Nanomedicine, Houston Methodist Research Institute, Houston, TX 77030, USA

**Keywords:** exosome, extracellular vesicle, microRNA, microvesicle, Toll-like receptor 4

## Abstract

Extracellular vesicles (EVs) are cell-released nanoparticles that transfer biomolecular content between cells. Among EV-associated biomolecules, microRNAs (miRNAs/miRs) represent one of the most important modulators of signaling pathways in recipient cells. Previous studies have shown that EVs from adipose-derived mesenchymal stromal cells (MSCs) and adipose tissue modulate inflammatory pathways in macrophages. In this study, the effects of miRNAs that are abundant in adipose tissue EVs and other biogenic nanoparticles (BiNPs) were assessed in terms of altering Toll-like receptor 4 (TLR4)-induced cytokines. TLR-4 signaling in macrophages is often triggered by pathogen or damage-induced inflammation and is associated with several diseases. This study demonstrates that miR-451a, which is abundant in adipose tissue BiNPs, suppresses pro-inflammatory cytokines and increases anti-inflammatory cytokines associated with the TLR4 pathway. Therefore, miR-451a may be partially responsible for immunomodulatory effects of adipose tissue-derived BiNPs.

## 1. Introduction

Extracellular vesicles (EVs) are biogenic nanoparticles (BiNPs) that are released by cells into the extracellular space, and can be found in all tissues and bodily fluids [1]. EVs are involved in (patho)physiological intercellular communication via the transportation of nucleic acids [2], proteins [3], lipids [4], and carbohydrates [5] to recipient cells. In many cases, the biophysical and molecular properties of EVs mediate cell and tissue-specific interactions, enabling targeted delivery of biomolecular cargo [6,7,8]. The ability of EVs to mediate site-specific delivery of endogenous and exogenous bioactive cargo make them promising for therapeutic applications [1]. In particular, EVs have emerged as a promising therapeutic modality for diseases involving inflammation, such as organ ischemia injury, graft-versus-host disease, liver failure, dermatological conditions, and chronic kidney disease [9,10,11,12,13,14]. The aforementioned therapeutic strategies rely on cultured cells, such as mesenchymal stromal cells (MSCs), as a source material for EVs. However, cell culture has many disadvantages, including low EV yields and expensive, time-consuming protocols for cell expansion [15]. The circumvention of cell culture by using EVs directly obtained from tissues and bodily fluids, including plasma and adipose tissue, is a time-efficient and cost-effective approach to obtain large quantities of EVs [15]. However, it is worth noting that isolation of EVs from tissue and bodily fluids usually results in contamination with other BiNPs, primarily lipoproteins [15]. Therefore, the term BiNP more accurately depicts the entire extracellular, nanosized content of adipose tissue.

Previously, it was shown that adipose tissue-derived BiNPs, including EVs, collected from patients undergoing liposuction, have protective and anti-inflammatory effects in macrophages [16]. Specifically, these BiNPs were shown to suppress the Toll-like receptor (TLR) 4 pathway, which is often triggered by pathogen or damage-induced inflammation [17]. Stimuli, such as infections, tissue injury, and the presence of foreign bodies or irritants can activate TLRs in immune cells, such as monocytes and macrophages, triggering the secretion of proinflammatory cytokines and chemokines [17]. TLR4 has been closely linked to the development of inflammatory cardiovascular diseases, type 2 diabetes mellitus, and cancer [18,19,20,21].

Among cargo molecules in EVs, microRNAs (miRNAs/miRs) are thought to be one of the primary mediators of physiological function [22,23,24]. These biomolecules are a class of endogenous, evolutionarily conserved, short non-coding RNAs that act post-transcriptionally as regulators of messenger RNA (mRNA) to protein translation. The first miRNA, lin-4, was originally discovered in Caenorhabditis elegans in 1993 by Lee and colleagues and was essential for lin-14 gene regulation in postembryonic developmental events [25,26]. After the discovery of lin-4, additional miRNAs have been found and investigated in most eukaryotes, including humans [27,28]. According to a recent release of miRBase (Release 22), 38,589 total and 1917 different human miRNAs have been reported to date [29]. It is predicted that more than 60% of all human protein-coding genes have been under selective pressure to maintain pairing to miRNAs [28]. Evidence highlights the pivotal roles of miRNAs in various biological and pathogenic processes, including inflammation. To date, several miRNAs related to the inflammatory response have been identified, such as miR-10, miR-146, miR-155, and miR-130b [30,31,32,33,34]. In this study, the effects of miRNAs that are abundant in adipose tissue BiNPs were assessed in terms of altering TLR4-induced cytokines in macrophages.

## 2. Materials and Methods

### 2.1. Cell Culture

The murine macrophage cell line (RAW264.7, American Type Culture Collection, Manassas, VA, USA), maintained in Dulbecco’s modified Eagle’s medium (DMEM) supplemented with 10% fetal bovine serum (FBS), 100 U/mL penicillin and 100 µg/mL streptomycin and cultured at 37 °C in a humidified atmosphere with 5% CO_2_.

The collection of residual de-identified subcutaneous adipose tissue from healthy, non-obese patients undergoing a liposuction procedure (lipoaspirate) was approved by the Mayo Clinic Biospecimens Review Group (ID:17-010290), and performed as previously described [16]. Human adipose-derived mesenchymal stromal cells (MSCs) were isolated from microfragmented adipose tissue samples, cultured in advanced minimum essential medium (MEM) supplemented with 5.35% PLTMax Human Platelet Lysate (MilliporeSigma, Burlington, MA, USA), 0.2% heparin (Sigma-Aldrich, St. Louis, MO, USA), 1% glutamax (Gibco, Thermo Fisher Scientific, Waltham, MA, USA), and 1% penicillin/streptomycin (Life Technologies, Carlsbad, CA, USA), and characterized as previously described [16]. MSC-EVs were isolated from conditioned cell culture media at passage four in medium without PLTMax and heparin using tangential flow filtration (TFF), as previously described and validated [16,35,36,37].

### 2.2. Lipoaspirate Processing

Lipoaspirate was obtained as outlined in the cell culture section and as previously described [16]. Following removal of the cell content, the liquid samples underwent TFF to obtain EVs and other BiNPs.

### 2.3. Nanoparticle Tracking Analysis (NTA)

The size distribution and particle concentration of isolated BiNPs were determined with NTA. Samples were diluted (1:100) in phosphate buffered saline and analyzed with a Nanosight NS300 (Malvern Panalytical, Malvern, United Kingdom), using the following measurement settings: three replicates with measurement time at 60 s.

### 2.4. Cryogenic Transmission Electron Microscopy (cryo-TEM)

Adipose tissue BiNPs and adipose-derived MSC EVs were imaged by cryo-TEM, as previously described [16]. Specimen preparation was carried out in a controlled environment vitrification system (CEVS) at 25 °C and 100% relative humidity. A small volume (3 µL) of BiNPs (10^10^/mL) was placed on a carbon-coated perforated polymer film, mounted on a 200 mesh TEM grid, as previously described [14]. To thin the drop into the film, excess solution was blotted away with a filter paper-covered metal strip. The grid was plunged into freezing liquid ethane (−183 °C), and transferred under liquid nitrogen into a Gatan 626 (Gatan, Pleasanton, CA, USA) cryo-holder. The grid was imaged at −175 °C in an FEI (now Thermo Fisher Scientific, Waltham, MA, USA) Talos 200C high-resolution TEM at an acceleration voltage of 200 kV. Images were recorded digitally by an FEI Falcon III. A Volta “phase-plate” that converts image phase differences into amplitude differences was used to enhance image contrast.

### 2.5. Western Blot Analysis

The protein content of the BiNPs was quantified using a bicinchoninic acid (BCA) assay (Thermo Fisher Scientific, Waltham, MA, USA). Equal amounts of sample protein were mixed with 6x reducing sodium dodecylsulfate (SDS) sample loading buffer (Boston Bioproducts, Ashland, MA, USA) and heated at 95 °C for 5 minutes. A control was prepared from MSCs lysed with radioimmunoprecipitation assay buffer (RIPA buffer, Thermo Fisher Scientific, Waltham, MA, USA). Proteins from samples (14.2 µg/well) were separated via gel electrophoresis using 4–12% Bis-Tris Plus gels (Invitrogen, Thermo Fisher Scientific, Waltham, MA, USA) and transferred to nitrocellulose membranes (Abcam, Cambridge, United Kingdom). After blocking in 5% milk (*w*/*v*) in Tris-buffered saline with 0.1% tween (TBST), the membranes were incubated with the following primary antibodies in 1% milk in TBST (*w*/*v*): anti-CD81 mouse monoclonal (1:500; Santa Cruz, Santa Cruz, CA, USA), anti-CD9 mouse monoclonal (1:500; Invitrogen, Thermo Fisher Scientific, Waltham, MA, USA), anti-CD63 rabbit monoclonal (1:500; Abcam, Cambridge, United Kingdom), anti-annexin V rabbit polyclonal (1:500; Abcam, Cambridge, United Kingdom), and anti-calnexin rabbit monoclonal (1:500; Abcam, Cambridge, United Kingdom). The membranes were incubated with the following secondary antibodies: rabbit polyclonal secondary antibody to mouse, and goat polyclonal secondary antibody to rabbit (all horseradish peroxidase, HRP-conjugated), at 1:3000 (Cell Signaling Technology, Danvers, MA, USA). Antibodies were detected by chemiluminescence (SuperSignal West Femto, Thermo Fisher Scientific, Waltham, MA, USA). Imaging analysis was performed with the myECL Imager (Thermo Fisher Scientific, Waltham, MA, USA).

### 2.6. Lipoprotein Quantification

The presence of lipoprotein contaminants, such as low-density lipoprotein (LDL), very-low-density lipoprotein (VLDL), and high-density lipoprotein (HDL) in BiNP samples (10^10^/mL) were assessed using a cholesterol assay kit (ab65390; Abcam, Cambridge, United Kingdom) according to the manufacturer’s instructions. Commercial LDL (ab91115; Abcam, Cambridge, United Kingdom) was used as a positive control.

### 2.7. RNA Next-Generation Sequencing (NGS)

The miRNA content of donor-matched adipose tissue BiNPs and adipose-derived MSC EVs was assessed by QIAGEN Genomic Services (Hilden, Germany) through NGS. RNA was isolated using the miRNeasy Serum/Plasma Kit according to the manufacturer’s instructions, and library preparation was done using the QIAseq miRNA Library Kit (Qiagen, Hilden, Germany). Total RNA (5 μL) was converted into miRNA NGS libraries and adapters containing unique molecular identifiers were ligated to the RNA. Then RNA was converted to cDNA and amplified using polymerase chain reaction (PCR) (22 cycles) and during the PCR indices were added. Samples were purified after PCR and library preparation quality control was performed using either TapeStation 4200 (Agilent, Santa Clara, CA, USA) or Bioanalyzer 2100 (Agilent, Santa Clara, CA, USA). Library pool(s) were quantified using quantitative PCR and sequenced on a NextSeq500 sequencing instrument according to the manufacturer’s instructions.

### 2.8. Quantitative Real-Time (qRT)-PCR

RAW 264.7 cells were seeded in 12-well plates in complete medium and transfected with a miR-451a mimic, miR-16-5p mimic, miR-451a inhibitor, or respective negative controls (NCs) (230 nM; Sigma-Aldrich, St. Louis, MO, USA) using Lipofectamine 3000 (Thermo Fisher Scientific, USA) according to the manufacturer’s instructions. After six hours, cells were washed with phosphate buffered saline (PBS) and stimulated with 500 ng/mL LPS (TLR4 ligand) for 4–24 h. Total RNA was isolated from RAW 264.7 cells with the RNeasy Plus Mini kit (Qiagen, Hilden, Germany) or from donor-matched adipose-derived MSC EVs and adipose tissue-derived BiNPs (100 µL) using the TRIzol reagent (Thermo Fisher Scientific, Waltham, MA, USA) with 25 pmol of synthetic cel-miR-39 (Qiagen, Hilden, Germany), which was used for normalization of RNA isolation and reverse transcription efficiency. RNA quantification and purity was assessed using NanoDrop 1000 spectrophotometer (Thermo Fisher Scientific, Waltham, MA, USA). RAW 264.7 mRNA for inflammatory cytokines (1 μg) and EV/BiNP miRNAs (100 ng) were reverse transcribed into the first-strand cDNA using the iScript cDNA synthesis kit (Bio-Rad Laboratories, Hercules, CA, USA). qRT-PCR analysis for Raw 264.7 cytokine mRNAs and BiNP miRNAs (miR-451a and miR-16-5p) was performed with a 7500 Fast Real-Time PCR System (Applied Biosystems, Waltham, MA, USA) using PowerUp SYBR Green Master Mix (Thermo Fisher Scientific, Waltham, MA, USA) according to the manufacturer’s instructions. Values were normalized to S26 and cel-miR-39/U6 according to the 2−ΔΔCt method in order to calculate the relative RAW 264.7 mRNA and EV/BiNP miRNA expression levels, respectively. All primers are listed in Table 1.

### 2.9. Enzyme-Linked Immunosorbent Assay (ELISA)

RAW 264.7 cells were seeded in 12-well plates in complete medium and transfected with a miR-451a mimic, miR-451a inhibitor, and respective negative controls (NCs) (350 nM; Sigma-Aldrich, St. Louis, MO, USA) using Lipofectamine 3000 (Thermo Fisher Scientific, Waltham, MA, USA) according to the manufacturer’s instructions. After six hours, cells were washed with PBS and stimulated with 500 ng/mL LPS (TLR4 ligand) for 48 h. EV/BiNP studies were performed as described above, with the exception of washing cells with PBS and adding EVs/BiNPs (10^9^/mL) three hours after transfection. Cell culture medium was collected, centrifuged (206× *g* at 4 °C for 10 min), aliquoted, and immediately stored at −80 °C. The medium was thawed on ice and IL-10 (#M1000B, R&D Systems, Minneapolis, MN, USA) and TNFα (#MTA00B, R&D Systems, Minneapolis, MN, USA) ELISAs were performed according to the manufacturer’s instructions. Absorbance at 450 nm was read with a microplate reader (SpectraMax M5, Molecular Devices, San Jose, CA, USA).

## 3. Results and Discussion

### 3.1. Characterization of Adipose-Derived MSC EVs and Adipose Tissue-Derived BiNPs

**Figure 1:**
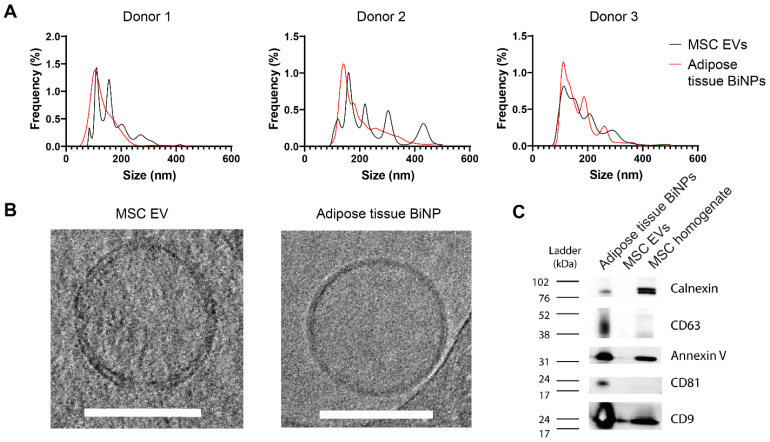
Characterization of donor-matched adipose-derived mesenchymal stromal cell (MSC) extracellular vesicles (EVs) and adipose tissue biogenic nanoparticles (BiNPs). (**A**) Size distribution determined by nanoparticle tracking analysis. (**B**) Morphological evaluation of EVs/BiNPs by cryogenic transmission electron microscopy. Scale bars, 100 nm. (**C**) Protein characterization of EVs/BiNPs by Western blot (samples were normalized to protein concentration). CD, cluster of differentiation.

Adipose tissue BiNPs were isolated from cell-depleted lipoaspirate, and adipose-derived MSC EVs were isolated from conditioned medium by TFF. BiNPs/EVs were characterized in accordance with the 2018 minimal information for studies of EVs (MISEV) guidelines [38]. NTA of donor-matched MSC EVs and adipose tissue BiNPs showed similar size distribution profiles ranging from 40–400 nm with most particles in the 80–280 nm range (Figure 1A). Cryo-TEM showed that BiNPs from both sources had a lipid bilayer, which is a key attribute for the authentication of EVs (Figure 1B).

Analysis of EV markers by Western blot revealed the presence of EV-associated transmembrane and cytoplasmic proteins, such as CD9 and annexin V (Figure 1C). The adipose tissue BiNPs displayed a greater variety and higher levels of EV markers compared to MSC EVs when normalized to protein content (Figure 1C). On the contrary, when samples were normalized according to particle number, markers on MSC EVs were enriched [16]. The particle/protein ratio is substantially higher for adipose tissue BiNPs [16], which is a likely explanation for this discrepancy. The intracellular contaminant marker, calnexin, was absent in MSC EVs, while the adipose tissue BiNPs showed much lower expression of calnexin compared to cell homogenate (Figure 1C). It is likely that a small proportion of cells were lysed during the lipoaspiration procedure, resulting in low levels of calnexin, which has also been shown in a previous study [35].

### 3.2. Adipose Tissue BiNPs Contain Higher Levels of miR-451a and miR-16-5p Compared to MSC EVs

**Figure 2:**
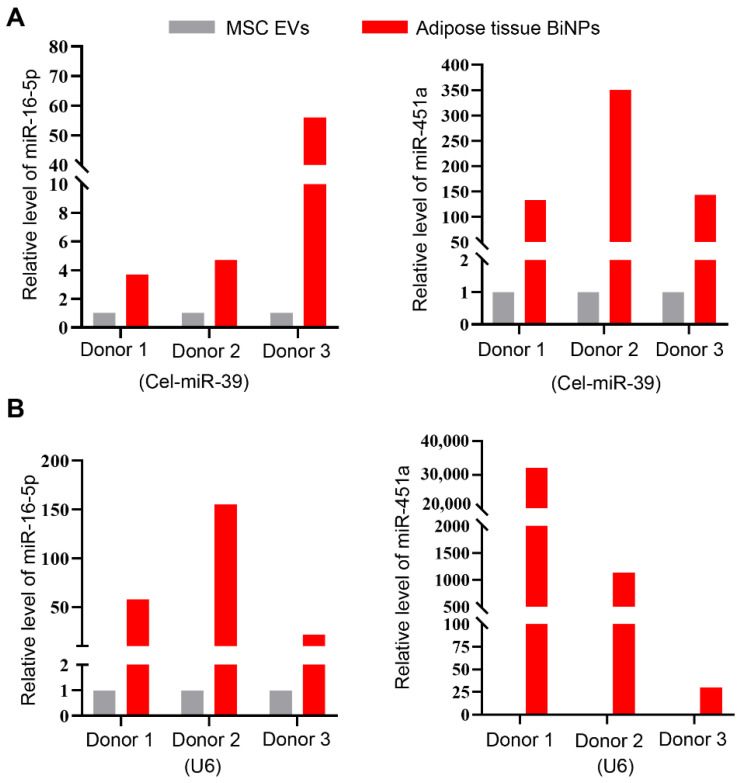
Quantitative real-time polymerase chain reaction (qRT-PCR) levels of microRNAs (miR)-16-5p and miR-451a in three donor-matched adipose-derived MSC EVs and adipose tissue BiNPs normalized for Cel-miR-39 (**A**) and U6 (**B**).

NGS results shed light on the ten most abundant miRNAs in adipose tissue BiNPs (Table 2). A literature search and gene ontology (GO) analysis of these top ten miRNAs revealed that miR-16-5p and miR-451a (among the three most highly expressed) have previously been shown to suppress TLR-4-induced inflammation in epithelial and microglial cells, respectively (Table 2). The relative abundance of miRNA-451a and miRNA-16-5p was much higher in adipose tissue BiNPs compared to donor-matched MSC EVs (Table 2). qRT-PCR validation demonstrated that this was the case for three different donor-matched BiNP/EV samples (Figure 2A,B).

**Figure 3:**
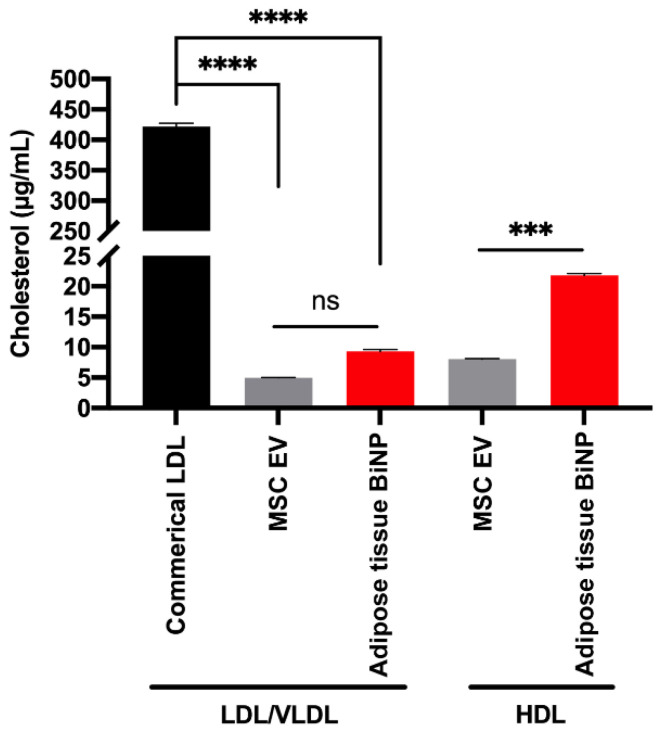
Cholesterol concentration associated with low-density lipoprotein (LDL) and very-low-density lipoprotein (VLDL) or high-density lipoprotein (HDL) in EV/BiNP samples. Commercial LDL at a concentration of 500 µg/mL apolipoprotein B-100 (ApoB-100) was used as a positive control (1000 µg/mL of ApoB-100 is the upper limit in the plasma of healthy individuals). Data are presented as mean ± standard deviation (SD) of three replicates. Statistics by analysis of variance (ANOVA) with Tukey’s multiple comparison post-hoc analysis. ***, *p* < 0.001; ****, *p* < 0001. ns, not significant.

It is important to note that EVs and lipoprotein overlap in size and density [39], making it very challenging to separate these two biogenic nanoparticles. Adipose tissue BiNP samples have previously been found to contain apolipoproteins (apo) [16]; however, recent studies have demonstrated that these proteins can be part of the EV protein corona [40], which does not necessarily indicate the presence of intact lipoproteins. Therefore, in this study, an assay that assesses LDL/VLDL cholesterol and HDL cholesterol was performed. The upper limit of apoB-100 (LDL protein component) in the plasma of healthy individuals is 1000 μg/mL [41], and is likely to be similar in the interstitial fluid of adipose tissue. Therefore, commercial LDL at half of this concentration (500 μg/mL apoB-100) was assessed for comparative purposes. The results indicate that the adipose tissue BiNP samples have two-fold higher LDL cholesterol levels compared to MSC EVs, although the difference is not statistically significant (Figure 3). The LDL cholesterol levels in adipose tissue BiNPs is 1% of the upper limit in the circulation of healthy individuals (Figure 3), indicating that the vast majority of these lipoproteins are removed during BiNP isolation. The adipose tissue BiNPs also displayed three-fold higher HDL cholesterol levels compared to MSC-EVs (Figure 3). MSCs do not produce LDL or HDL, so the residual levels detected in the assay may be cholesterol associated with the lipid bilayer of EVs, or alternatively, lipoproteins that were internalized by cells during earlier passages when exposed to platelet lysate, and later secreted into the medium (cells were grown without platelet lysate for the EV collection passage). Taken together, it is possible that miRNA-451a and miRNA-16-5p in the adipose tissue samples are associated with both EVs and lipoproteins. Nevertheless, the samples lack smaller miRNA complexes, as tangential flow filtration is an effective method for isolating particles in the size range of 60–600 nm [35]. It is worth nothing that the detected miR-451a and miR-16-5p could be associated with EVs in various ways, as it is currently unknown to what extent miRNAs are present in the EV interior versus being bound to the exterior surface [42]. In any case, detected miRNAs are protected from enzymatic degradation, as ribonuclease activity is high in interstitial fluid [43].

The two miRNAs (miRNA-451a and miRNA-16-5p) were selected for further assessment of TLR4 effects in macrophages, as adipose tissue BiNPs have previously been shown to suppress TLR4-induced cytokine secretion in these cells [16]. Additionally, nanosized particles, including EVs, accumulate primarily in macrophages upon intravenous administration [14,44,45,46,47], indicating the importance of studying the effects of BiNPs on this cell type.

### 3.3. Effects of miR-451a and miR-16-5p on the Expression of Inflammatory Cytokines in LPS-Stimulated Macrophages

**Figure 4:**
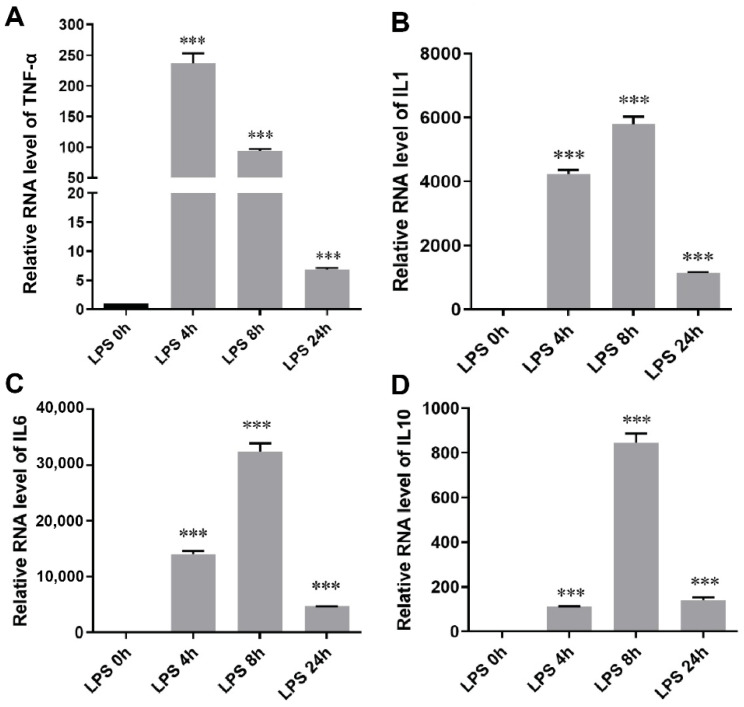
RNA expression levels of tumor necrosis factor-α (TNF-α) (**A**), interleukin (IL)-1β (**B**), IL-6 (**C**), and IL-10 (**D**) in RAW 264.7 macrophages exposed to 500 ng/mL lipopolysaccharide (LPS). Data are presented as mean ± standard error of mean (SEM) of three replicates. Statistics by ANOVA with Tukey’s multiple comparison post-hoc analysis. ***, *p* < 0.001.

Macrophages play a central role in inflammation by releasing proinflammatory cytokines and other soluble inflammatory mediators, such as tumor necrosis factor alpha (TNF-α), which can activate endothelial cells [59,60]. The anti-inflammatory effects of adipose tissue BiNP-enriched miRNAs (miR-451a and miR-16-5p) on inflammatory responses in LPS (TLR4 ligand)-induced macrophages were assessed using lipofectamine transfected mimics (chemically synthesized). Previous studies have demonstrated that the effects of adipose tissue BiNPs in endotoxin-stimulated primary human macrophages (differentiated from monocytes) are similar to those in RAW 264.7 macrophages [16], therefore, the latter cell line was used in this study for practical reasons. Optimization studies in RAW 264.7 cells were performed to evaluate which cytokines are most highly elevated in response to LPS. Based on these optimization studies, TNF-α, interleukin (IL)-1β, IL-6, and IL-10 were selected secondary to robust TLR4-induced cytokine expression (Figure 4A–D).

**Figure 5:**
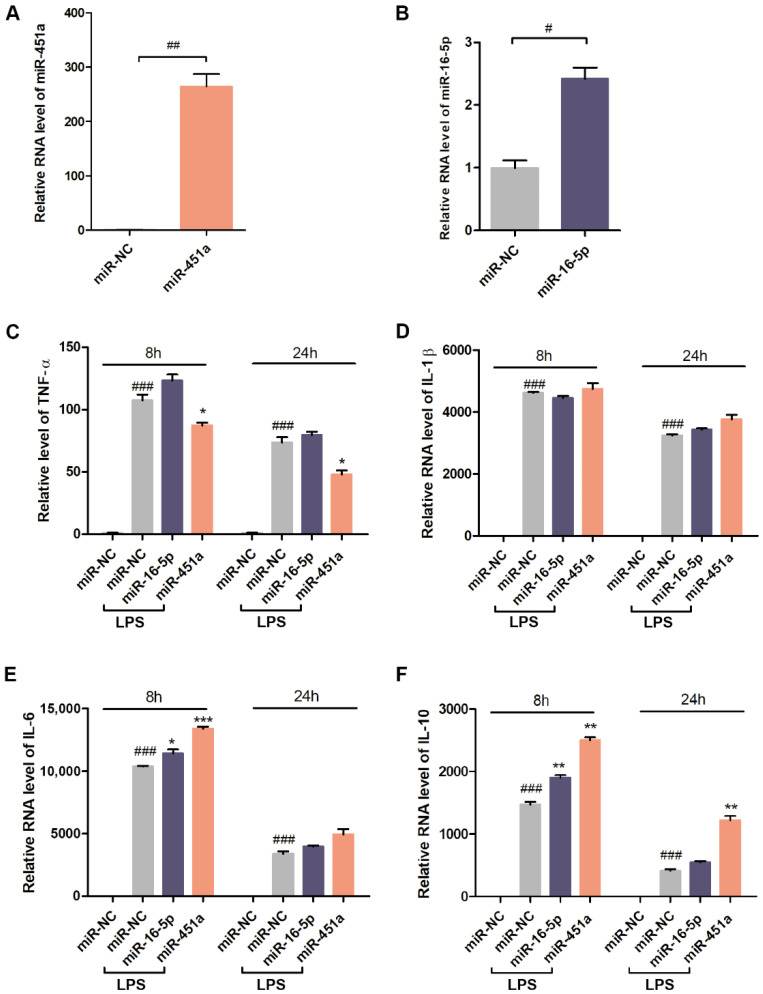
Effects of miR-451a and miR-16-5p synthetic mimics on Raw 264.7 macrophages. (**A**,**B**) Intracellular levels of miR-451a and miR-16-5p after transfecting cells with miRNA mimics. (**C**–**F**) RNA expression levels of TNF-α, IL-1β, IL-6, IL-10 in the presence of miR-451a and miR-16-5p mimics in cells exposed to LPS. Data are presented as mean ± SEM of three replicates. Statistics by ANOVA with Tukey’s multiple comparison post-hoc analysis. ^#^, *p* < 0.05; ^##^, *p* < 0.01; ^###^, *p* < 0.001 in comparison to miR negative control (NC) without LPS unless otherwise indicated. *, *p* < 0.05; **, *p* < 0.01, ***, *p* < 0.001 in comparison to miR-NC with LPS.

The presence of intracellular miR-451a and miR-16-5p mimics following transfection was verified by qRT-PCR (Figure 5A,B). The inflammatory cytokine, TNF-α, was significantly reduced at both time points upon exposure to miR-451a, while miR-16-5p did not alter the levels of this cytokine (Figure 5C). The levels of LPS-induced IL-1β remained unchanged in response to both mimics (Figure 5D), while IL-6 was slightly increased by both mimics at the shorter time point (eight hours) (Figure 5E). The miR-451a mimic substantially increased the anti-inflammatory cytokine, IL-10, at both time points, while the miR-16-5p mimic did so to a lesser extent at the eight-hour time point (Figure 5F). These results demonstrate that miR-451a suppresses inflammatory cytokine secretion (TNF-α) caused by the TLR4 activation and increases anti-inflammatory cytokine secretion (IL-10); whereas miR-16-5p has minimal effects on LPS-induced cytokine secretion in RAW 264.7 cells. It is worth noting that the lack of miR-16-5p effects could be due to the modest increase in expression (2.5-fold) compared to control cells. Previous studies have shown that miR-16-5p mimics typically result in a modest 1.5-3.5-fold increase in expression in various cell lines [61,62,63,64], which is likely to be due to relatively high endogenous expression of this miR.

### 3.4. Effects of miR-451a Inhibition on IL-10 and TNF-α in LPS-Stimulated Macrophages Treated with MSC EVs and Adipose Tissue BiNPs

**Figure 6:**
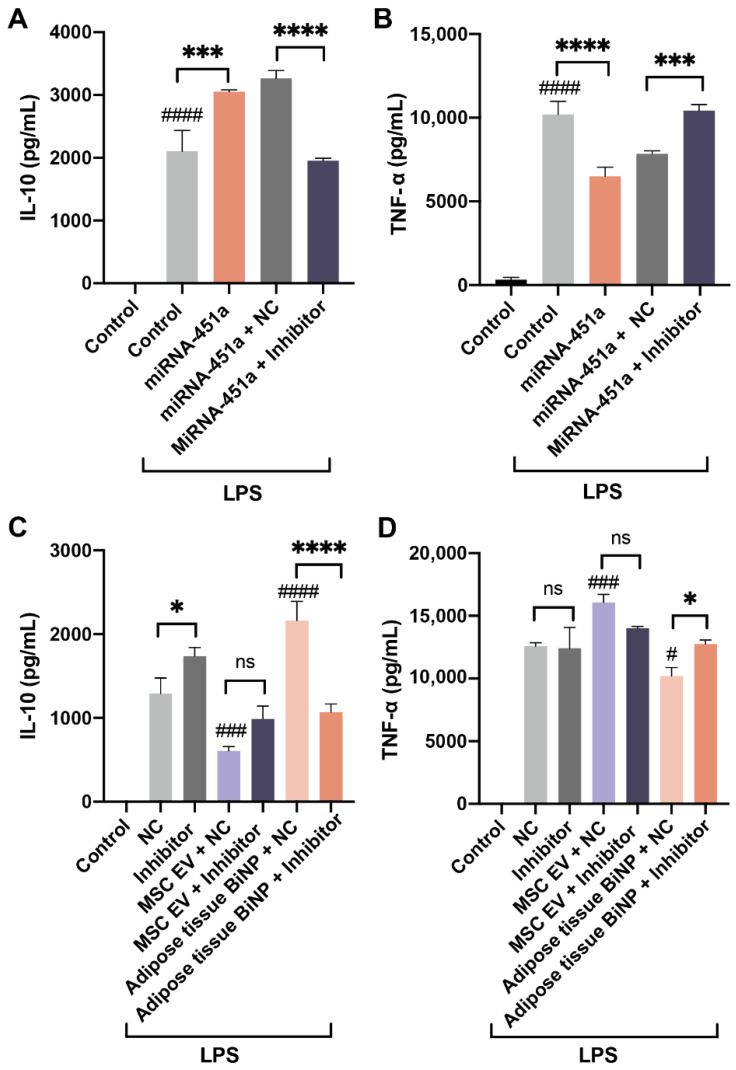
Effects of a miR-451a steric blocking oligonucleotide inhibitor on protein levels of IL-10 and TNF-α in RAW 264.7 macrophages exposed to LPS (500 ng/mL). Effects of co-exposure to the miR-451a inhibitor and a miR-451a synthetic mimic on IL-10 (**A**) and TNF-α (**B**) expression. Effects of co-exposure to the miR-451a inhibitor and donor-matched MSC EVs or adipose tissue BiNPs (10^9^/mL) on IL-10 (**C**) and TNF-α (**D**) expression. Data are presented as mean ± SD of three replicates. Statistics by ANOVA with Tukey’s multiple comparison post-hoc analysis. ^#^, *p* < 0.05; ^###^, *p* < 0.001; ^####^, 0.0001 in comparison to control without LPS (**A**,**B**) or NC (**C**,**D**). *, *p* < 0.05; ***, *p* < 0.001, ****, *p* < 0.0001.

Previous studies have demonstrated that MSC EVs and adipose tissue BiNPs can modulate inflammatory pathways in macrophages [16,65,66]. The contribution of miR-451a to BiNP effects on LPS-stimulated macrophages was assessed using a potent steric blocking oligonucleotide inhibitor of miR-451a. Similar to the qRT-PCR studies, the miR-451a mimic increased protein levels of IL-10 (Figure 6A) and decreased protein levels of TNF-α in LPS-treated RAW 264.7 cells (Figure 6B). The observed miR-451a mimic-induced increase in IL-10 and decrease in TNF-α were reversed in the presence of a miR-451a steric blocking oligonucleotide inhibitor (Figure 6A,B).

Cells were then treated with BiNPs and the miR-451a inhibitor to assess effects on IL-10 and TNF-α. The results revealed that the levels of IL-10 decreased when cells were exposed to adipose tissue BiNPs in the presence of the miR-451a inhibitor, while IL-10 levels in cells exposed to MSC EVs did not change significantly in the presence of the inhibitor (Figure 6C). A similar effect was observed with TNF-α levels, where the miR-451a inhibitor reversed the effects of adipose tissue BiNPs, but failed to cause a significant change in cells exposed to MSC EVs (Figure 6D). Taken together, these results indicate that miR-451a plays a role in adipose tissue BiNP-mediated effects in TLR4-stimulated macrophages. On the contrary, the inhibitor did not cause significant changes in cells treated with donor-matched MSC EVs, which is most likely due to miR-451 being much less abundant in these EVs.

## 4. Conclusions

Inflammation is an essential immune response to injury and infection contributing to the protection of the body and tissue healing. However, excessive/chronic inflammatory responses are detrimental to tissue function and have been associated with many severe diseases [67]. While the inflammatory response involves complex signaling pathways with several potential points of regulation, research in the past decade has emphasized the potential of TLRs as therapeutic targets [17]. Two of the most highly expressed miRNAs in adipose tissue BiNPs, miR-451a and miR-16-5p, have previously been found to suppress TLR4-induced cytokines in microglial cells [49] and lung epithelial cells [48], respectively. The goal of this study was to assess the effects of these two miRNAs on TLR4-mediated cytokine secretion in macrophages and to determine whether they could be partially responsible for adipose tissue BiNP-mediated immunomodulatory effects. The results indicate that the presence of miR-451a produces a significant suppression of an LPS-induced inflammatory cytokine (TNF-α) and a significant increase in an anti-inflammatory cytokine (IL-10), while miR-16-5p does not have any substantial effects on cytokine expression. The use of a potent steric inhibitor reversed the effects of the miR-451a mimic and altered the anti-inflammatory effects of adipose tissue BiNPs. The inhibitor failed to significantly alter the effects of donor-matched MSC EVs, which contain substantially lower levels of miR-451a. Although both MSC EVs and adipose tissue BiNPs have been shown to modulate inflammatory pathways in macrophages [16,65,66], these effects are likely to be mediated by distinct biomolecular cargo.

BiNPs from fresh adipose tissue are attractive for clinical applications, as isolation is easier, faster, and more cost-efficient compared to MSC-derived EVs [15]. Anti-inflammatory effects mediated by BiNP-associated miR-451a could be beneficial for prevention of ischemia-reperfusion injury of organs, including donor organs used for transplantation [12]. Other potential clinical applications include attenuation of neointimal formation after vascular injury to prevent in-stent restenosis, and other occlusive vascular diseases [68]. The results of this study warrant further investigation into miR-451a as a mediator of immunomodulatory effects of adipose tissue BiNPs.

## Figures and Tables

**Table 1:** Primers used in the study.

Primers	Forward Sequences (5′→3′)	Reverse Sequences (5′→3′)
IL-1β	TACCTGTCCTGCGTGTTGAA	TCTTTGGGTAATTTTTGGGATCT
IL-6	CCTCTGGTCTTCTGGAGTACC	ACTCCTTCTGTGACTCCAGC
IL-10	GCTCTTGCACTACCAAAGCC	CTGCTGATCCTCATGCCAGT
TNF-α	AGCCCCCAGTCTGTATCCTT	GAGGCAACCTGACCTCTCTC
S26	TCATTCGGAACATTGTAGAAGCC	AGCTTGACATAGAGCTTGGGAA
miR-451a RT	GTCGTATCCAGTGCAGGGTCCGAGGTGCACTGGATACGAC AACTCAG	
miR-451a	TGCGGAAACCGTTACCATTACTG	CCAGTGCAGGGTCCGAGGT
miR-16-5p RT	GTCGTATCCAGTGCAGGGTCCGAGGTGCACTGGATACGACCGCCAAT	
miR-16-5p	TGCGGTAGCAGCACGTAAATATT	CCAGTGCAGGGTCCGAGGT
U6 RT	GTCGTATCCAGTGCAGGGTCCGAGGTATTCGCACTGGATACGACAAAATATGGAAC	
U6	TGCGGGTGCTCGCTTCGGCAGC	CCAGTGCAGGGTCCGAGGT
Cel-miR-39 RT	GTCGTATCCAGTGCAGGGTCCGAGGTGCACTGGATACGACCAAGCTG	
Cel-miR-39	TGCGGTCACCGGGTGTAAATCAG	CCAGTGCAGGGTCCGAGGT

**Table 2:** Ten most abundant microRNAs (miRNAs/miRs) in adipose tissue biogenic nanoparticles (BiNPs) and fold-changes compared to donor-matched adipose-derived mesenchymal stromal cell (MSC) extracellular vesicles (EVs) based on trimmed mean of the M-values (TMM) normalization of next-generation sequencing counts.

miRNAs (Listed According to Abundance)	Suppression of Toll-like Receptor 4 (TLR-4) Pathways		
	Fold-Change (Compared to MSC EVs)	References	Gene Ontology (GO) Analysis
miR-16-5p	11.6	Yes (lung epithelial cells) [48]	Yes
let-7a-5p	1.2	Unknown	Yes
miR-451a	179.7	Yes (microglial cells, lung tissue) [49,50]	Unknown
miR-143-3p	5.1	Yes (lung tissue) [51]	Unknown
let-7b-5p	1.0	Yes [52,53]	Yes
let-7f-5p	2.9	Unknown	Yes
miR-125b-5p	0.9	Yes (myoblast cells) [54]	Yes
miR-26a-5p	2.8	Yes (endothelial cells, microglial cells) [55,56]	Yes
let-7i-5p	0.9	Yes (endothelial cells, kidney epithelial cells, kidney tissue) [57,58]	Yes
miR-486-5p	29.2	Unknown	Unknown

## Data Availability

Data is contained within the article or from the authors upon reasonable request.
